# Advances in deer antler research based on single-cell multi-omics approaches

**DOI:** 10.3389/fcell.2026.1873515

**Published:** 2026-08-17

**Authors:** Hengxing Ba, Chunyi Li

**Affiliations:** Institute of Antler Science and Product Technology, Changchun Sci-Tech University, Changchun, China

**Keywords:** antler, hybrid ossification, rapid growth, regeneration, single-cell multi-omics, stem cells

## Abstract

Deer antlers are the only mammalian appendages capable of complete periodic regeneration and exceptionally rapid growth, providing a unique model for studying organ regeneration, tissue growth, and regenerative medicine. Recent advances in single-cell and spatial multi-omics technologies have substantially advanced our understanding of the cellular and molecular mechanisms underlying antler full regeneration and rapid growth. In this review, we summarize these advances by integrating transcriptomic, epigenomic, and spatial profiling approaches, with a focus on the identification of antler stem/progenitor cell populations, regulatory programs, osteochondral differentiation trajectories, and vascular remodeling that support rapid tissue expansion. We further discuss cross-species comparisons. Finally, we outline current challenges and future directions. These advances provide important insights into the mechanisms underlying mammalian appendage regeneration and rapid tissue growth, offering potential strategies for regenerative medicine and tissue engineering.

## Introduction

Deer antlers provide a remarkable example of mammalian organ regeneration, offering valuable insights for regenerative medicine. Antlers are male secondary sexual characteristics in cervids (excluding reindeer) and are unique among mammals as appendages capable of complete periodic regeneration (Landete-Castillejos et al., 2019; Li et al., 2024; Kierdorf et al., 2007). This remarkable regenerative capacity is orchestrated by antler stem cells (AnSCs), which express canonical mesenchymal stem cell markers as well as certain embryonic stem cell markers, conferring them with extraordinary proliferative and differentiation potential (Ba et al., 2019; Wang et al., 2019; Li et al., 2009b). *In vitro*, AnSCs can be continuously passaged for over 55 generations without senescence (Lei et al., 2021), They possess the capacity for differentiation toward multiple mesenchymal cell types, including chondrocytes, osteoblasts, and adipocytes (Wang et al., 2019; Li et al., 2009b). These findings support the mesenchymal multipotency of AnSCs; however, whether they possess broader lineage potential beyond mesenchymal derivatives remains to be determined.

**FIGURE 1:**
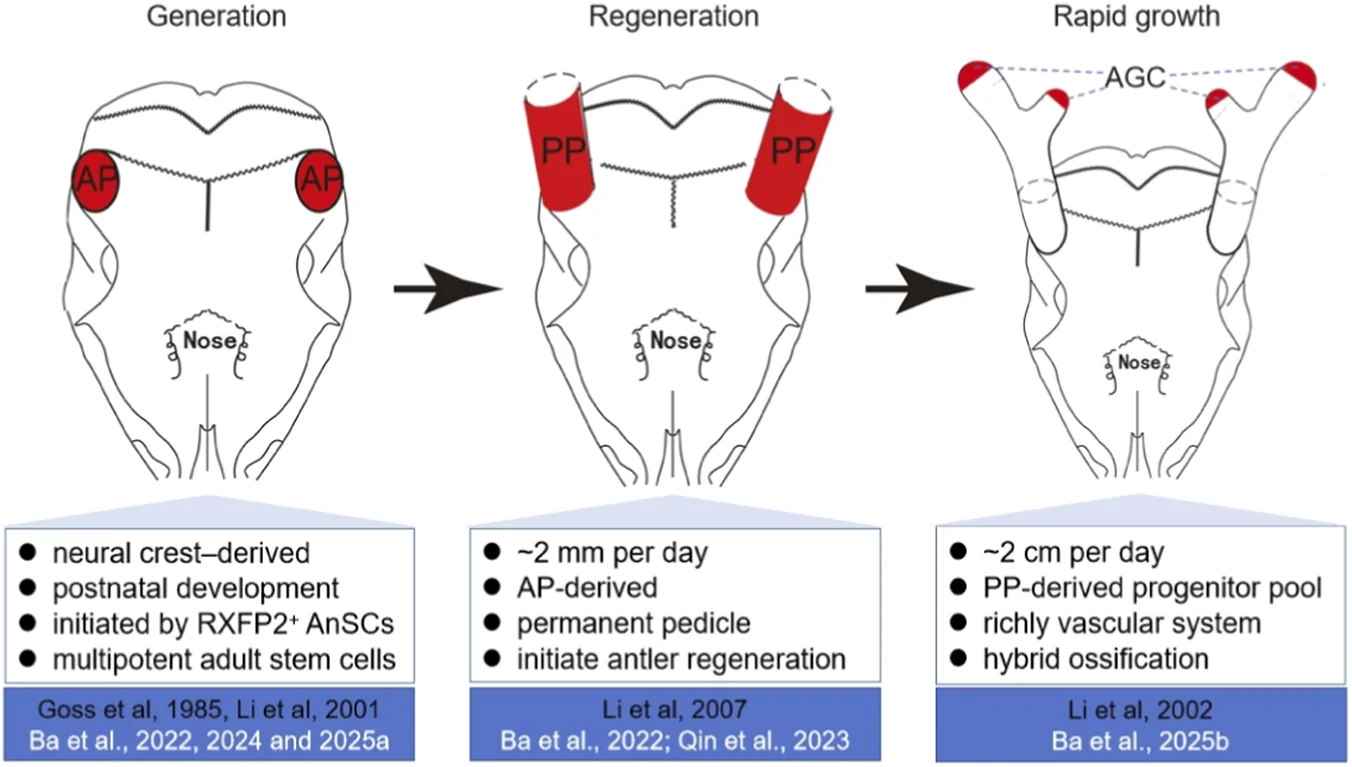
Schematic overview of antler generation, regeneration, and rapid growth. The associated tissues across different developmental stages are highlighted in red, and their key characteristics are summarized. Black references represent tissues identified through previous morphological and histological studies, whereas white references indicate tissues and cell populations that have been recently characterized by single-cell or spatial multi-omics studies. AP, antlerogenic periosteum; PP, pedicle periosteum; AGC, antler growth center; AnSCs, antler stem cells.

AnSCs are initially localized within the antlerogenic periosteum (AP) of the deer frontal crest region. At puberty, androgen signaling activates these resident AnSCs to initiate antlerogenesis, leading to the development of pedicles and the first antlers (a process termed antler generation), which subsequently enter an annual cycle of complete regeneration (Li and Suttie, 2001; Goss and Powel, 1985). Transplantation studies further demonstrate that AP possesses full organogenic potential, as it can induce ectopic or interspecies antler formation when grafted into nude mice or deer (Li et al., 2001; Li et al., 2008; Li et al., 2009a). Antler regeneration depends on the pedicle periosteum (PP) (Li et al., 2007; Kierdorf and Kierdorf, 2011), whose cells are directly derived from the AP. PP cells retain the capacity to sustain periodic antler regeneration and further differentiate into the antler growth center (AGC), a specialized structure responsible for rapid antler elongation. The distal AGC drives antler growth at rates of up to 2 cm per day, generating tens of kilograms of tissue within 3-4 months without tumor formation (Figure 1) (Li et al., 2023; Li et al., 2002).

Historically, antler research was primarily investigated through morphological and histological approaches (Goss and Powel, 1985; Kierdorf and Kierdorf, 2011; Landete-Castillejos et al., 2019; Li, 2023; Li et al., 2005; Li and Suttie, 2012), followed by the discovery and characterization of antler stem cells (Li et al., 2001; Li et al., 2008; Li et al., 2009a), which established the intrinsic organogenic competence of the AP (Li et al., 2009a; Li et al., 2001). However, the cellular heterogeneity, lineage relationships, and regulatory mechanisms underlying this remarkable regenerative and rapid growth capacity remained largely unresolved. Recent advances in single-cell and spatial multi-omics technologies have fundamentally shifted the conceptual framework of antler formation from a tissue-autonomous process toward a dynamic multicellular regenerative ecosystem (Ba et al., 2022; Qin et al., 2023; Ba et al., 2025a; Ba et al., 2025b). By resolving cellular identities, developmental trajectories, gene regulatory networks, and spatial organization, single-cell multi-omics approaches have revealed that antler generation, regeneration, and rapid growth are not driven solely by stem/progenitor cells, but depend on coordinated interactions among multiple cellular populations, including stem/progenitor cells, chondrocytes, vascular cells, and immune cells. These studies have advanced our understanding of mammalian appendage regeneration by uncovering how cellular interactions, molecular pathways, and tissue microenvironments collectively support regeneration and rapid tissue growth.

## Single-cell atlas of the antler

Early studies established the first single-cell atlas of activated and dormant AP, PP, and the adjacent non-regenerative facial periosteum, identifying diverse cell populations, including THY1^+^ AnSCs, TNN^+^TNC^+^DLX5^+^SOX9^+^RUNX2^+^ progenitor cells, osteochondroblasts, perivascular cells, endothelial cells, and immune cells (Ba et al., 2022). This work established the first comprehensive cellular atlas of antler generation and regeneration, providing a foundation for subsequent mechanistic studies of the cellular interactions and regulatory networks that govern these processes.

Subsequent single-cell analyses spanning multiple stages of antler regeneration further refined this framework. PRRX1^+^ AnSCs were identified as a major mesenchymal stem cell population activated during the earliest stages of antler regeneration, exhibiting transcriptional trajectories consistent with differentiation toward TNN^+^TNC^+^DLX5^+^ antler blastema progenitor cells (ABPCs), which represent a predominant progenitor population within the regenerative blastema (Qin et al., 2023). These findings support the concept that specialized mesenchymal stem/progenitor cell populations represent a key cellular reservoir contributing to antler regeneration. However, because definitive genetic lineage-tracing approaches and genetically engineered deer models are currently unavailable, most lineage relationships have been inferred from transcriptomic similarity and computational trajectory analyses. Therefore, the developmental potential and lineage boundaries of antler stem/progenitor cells require further functional validation.

More recently, spatial transcriptomics studies have provided a comprehensive view of the cellular organization and spatial architecture of the AGC during rapid antler growth (Ba et al., 2025a). Several late-stage developmental cell populations, including chondrocytes, hypertrophic chondrocytes, intermediate PHEX^+^ cells, osteoblasts, and osteoclasts, were identified within distinct spatially organized tissue layers. Spatial analyses further revealed a highly ordered distal-proximal developmental axis, highlighting the coordinated expansion, maturation, and differentiation of multiple tissue compartments during rapid antler growth.

## Signaling pathways regulating THY1^+^PRRX1^+^RXFP2^+^ AnSCs during antler generation

Further comparative single-cell transcriptomic analyses of periosteal tissues, including AP, facial periosteum, and long bone periosteum, identified a distinct antler stem cell subpopulation, THY1^+^PRRX1^+^RXFP2^+^ AnSCs, specifically enriched in the AP of both male and female deer (Ba et al., 2025b). Current single-cell transcriptomic studies support a model in which these THY1^+^PRRX1^+^RXFP2^+^ AnSCs represent a key stem cell subpopulation that gives rise to TNN^+^TNC^+^DLX5^+^SOX9^+^RUNX2^+^ antler progenitor cells, which subsequently differentiate into chondrogenic lineages (Ba et al., 2022; Qin et al., 2023; Han et al., 2025; Zhang and Xing, 2025). Emerging evidence suggests that multiple signaling pathways contribute to the activation and differentiation of THY1^+^PRRX1^+^RXFP2^+^ AnSCs. *In vitro* studies demonstrated that androgen treatment upregulates RXFP2 expression (Ba et al., 2022), while puberty-associated androgen signaling contributes to antler generation, suggesting a potential AR-RXFP2 regulatory axis in early antler generation. Independent evidence also implicates canonical Wnt signaling. Elevated expression of the Wnt co-receptor LRP6, together with impaired osteogenic differentiation following LRP6 knockout, suggests that LRP6-mediated Wnt signaling contributes to the activation and differentiation of THY1^+^PRRX1^+^RXFP2^+^ AnSCs (Ba et al., 2025b). Whether AR-RXFP2 and Wnt signaling act cooperatively or independently remains unclear, and the precise roles of these pathways require further validation through *in vivo* functional approaches.

## Immune regulation of antler generation

Accumulating evidence highlights the critical role of immune regulation in antler generation. IL-34 secreted by AnSCs in the male AP, but not in the female AP, has been shown to promote the recruitment of M2 macrophages. In xenogeneic antler formation assays using immunodeficient mice, inhibition of M2 macrophage infiltration markedly impaired antler formation efficiency, suggesting that immune–stem cell interactions play an important role in supporting antler generation (Ba et al., 2025b). Consistently, local administration of macrophage-derived factors was sufficient to induce antler generation in female deer (Wang et al., 2025), further supporting the notion that macrophage-associated signals participate in activating antler generation.

Single-cell transcriptomic analyses of reindeer antler-associated skin (velvet) further revealed a specialized immune microenvironment associated with scar-free regeneration (Sinha et al., 2022). Compared with dorsal skin, velvet fibroblasts exhibited fetal-like transcriptional features and immunomodulatory properties that restrict excessive inflammatory responses and favor regenerative outcomes over fibrosis.

These findings have expanded the traditional stem/progenitor cell-centric view of antler generation toward a multicellular ecosystem, where immune cells act as active regulators of the stem cell niche rather than merely passive participants. However, the molecular mechanisms by which immune-derived signals modulate AnSC activation, self-renewal, and lineage commitment remain largely unknown.

## Gene regulatory programs underlying rapid antler growth

Integrated chromatin accessibility and transcriptomic analyses further revealed distinct regulatory programs associated with rapid antler growth. Proliferative antler progenitor cells displayed enhanced expression and chromatin accessibility of genes involved in TP53 signaling and apoptosis-related pathways, whereas osteosarcoma cells preferentially activated DNA repair and MYC-driven transcriptional programs (Ba et al., 2025a). These findings suggest that rapid antler growth is not simply driven by enhanced proliferative activity but is supported by coordinated regulation of cell-cycle progression, stress responses, and genome stability maintenance.

Interestingly, MYC target genes in proliferative antler progenitor cells differed markedly from those observed in osteosarcoma cells, suggesting that antler growth utilizes a distinct transcriptional program that enables robust proliferation while maintaining tight control of cell fate and cellular homeostasis. Unlike malignant proliferation, in which MYC activation is frequently associated with uncontrolled growth and genomic instability, proliferative antler progenitor cells appear to employ regulatory mechanisms that balance proliferative capacity with tissue organization. These findings highlight the importance of integrating chromatin accessibility with transcriptomic information to identify rapid growth-specific gene regulatory networks. Nevertheless, whether how MYC cooperates with other transcription factors and epigenetic regulators to coordinate antler rapid growth, remain to be experimentally validated.

## PHEX^+^ intermediate cell population and hybrid ossification during rapid antler growth

Spatial transcriptomics identified a distinct PHEX^+^ cell population located between hypertrophic chondrocytes and osteogenic cells, whereas pseudotime analyses suggest a continuous transition from hypertrophic chondrocytes toward osteoblast-like cells through an intermediate PHEX^+^ cells (Ba et al., 2025a). In addition, xenotransplantation experiments provide supportive evidence for the potential contribution of hypertrophic chondrocyte-derived cells to osteogenic populations *in vivo*. These findings suggest that cellular fate transitions contribute to antler ossification. Accordingly, we propose a “hybrid ossification” model, in which hypertrophic chondrocyte-associated osteogenic transition may jointly contribute to bone formation during rapid antler growth. This model is currently supported by spatial transcriptomics and transplantation studies but has not yet been experimentally validated through direct lineage-tracing approaches. It is consistent with accumulating evidence from mammalian skeletal development showing that hypertrophic chondrocytes can contribute to osteoblast formation during endochondral ossification (Aghajanian and Mohan, 2018; Wolff and Hartmann, 2019; Zhou et al., 2014). Nevertheless, the identity of the PHEX^+^ intermediate population and the extent of chondrocyte-to-osteoblast conversion during rapid antler growth remain unresolved. Because genetic lineage-tracing systems and transgenic deer models are not yet available, direct evidence for lineage conversion is still lacking. Future studies integrating lineage-tracing strategies will be required to determine whether PHEX^+^ cells represent bona fide osteogenic intermediates or transient transcriptional states within a broader differentiation continuum.

## Vascularized cartilage and angiogenic niche formation during rapid antler growth

Unlike typical mammalian growth cartilage, the AGC contains a highly vascularized cartilage structure that is associated with exceptionally rapid tissue expansion (Clark et al., 2006). Integrated single-cell and spatial transcriptomic analyses revealed distinct spatial heterogeneity of vascular organization along the distal-proximal axis of the AGC (Ba et al., 2025a). The proximal region exhibited hypoxic and angiogenic signatures, characterized by elevated expression of HIF1A and VEGFA, whereas distal regions showed enrichment of vascular maturation-related genes, including PDGFB and ANGPT1. Cell-cell communication analyses further revealed that distal AGC regions interact with vascular cells through multiple angiogenic pathways, including PTN-SDC1, MDK-SDC1, WNT5A-FZD, and VEGFA-KDR, suggesting that coordinated vascular remodeling may support rapid antler growth.

Further evidence for the unique vascular niche comes from xenotransplantation studies, in which AP tissues grafted into immunodeficient mice formed a chimeric vascular system. Endothelial cells were mainly host-derived, whereas perivascular cells were associated with graft-derived progenitor populations (Wang et al., 2022). These findings suggest coordinated interactions between progenitor cells and host vasculature during vascular niche formation. The newly established vascular network may further support rapid antler growth by providing oxygen, nutrients, and angiogenic signals. Thus, antler stem/progenitor cells and vascular cells likely form a reciprocal regulatory network, in which vascular remodeling supports progenitor activity and osteochondral regeneration within the AGC.

## Cross-species comparisons

Comparative single-cell analyses across regenerative and non-regenerative systems have provided important insights into conserved and divergent cellular programs underlying epimorphic regeneration. During early antler regeneration (day 5 post-shedding), a distinct population of ABPCs emerges, expressing chondrogenic and limb development-associated genes, including PTN, TNC, TNN, and DLX5, with similar transcriptional programs also observed in regenerative mouse digit tip (P3) tissues but largely absent in non-regenerative P2 tissues (Qin et al., 2023). Although these mammalian developmental programs show limited overlap with other regenerative systems, including axolotl limb (Gerber et al., 2018; Leigh et al., 2018) and zebrafish fin regeneration (Jiang et al., 2021; Hou et al., 2020), the extent of their molecular and functional conservation remains unclear. These observations suggest that mammalian epimorphic regeneration may rely on the selective reactivation of developmental and regenerative programs, rather than the deployment of a universal regeneration-specific cell type or transcriptional module (Tajer et al., 2023; Min and Whited, 2023). Importantly, these findings should not be interpreted as evidence that blastema cells represent universally multipotent regenerative cells. Instead, accumulating evidence from diverse regenerative models indicates that blastemas are heterogeneous and dynamic structures composed of lineage-biased progenitors, transitional cell states, and niche-supporting populations (Min and Whited, 2023; Zhong et al., 2023). Therefore, antler blastema formation is more likely to reflect the coordinated reactivation of developmental and regenerative programs within a specialized cellular ecosystem, rather than a complete reversion to a pluripotent embryonic state.

In addition, antler stem/progenitor cell developmental origin represents another important axis for cross-species comparison. Neural crest-derived mesenchymal cells, often referred to as a highly multipotent and migratory cell lineage in vertebrate development, contribute to a broad range of craniofacial structures, including cartilage and bone (Shyamala et al., 2015; Soldatov et al., 2019). Both dental pulp mesenchymal stem cells and antler stem/progenitor cells have been proposed to originate, at least in part, from cranial neural crest-derived lineages. Recent single-cell transcriptomic studies further indicate transcriptional similarities between these cell populations, suggesting the existence of shared regulatory programs underlying craniofacial mesenchymal plasticity (Ba et al., 2024). However, whether these similarities reflect conserved developmental origins or convergent transcriptional states associated with regenerative capacity remains to be determined.

## Perspectives and future directions

In this review, single-cell multi-omics have been applied to systematically dissect the cellular and molecular mechanisms underlying antler generation, regeneration and rapid growth. These studies have substantially advanced our understanding of stem/progenitor cell heterogeneity, lineage specification, vascular niche formation, and intercellular communication. Nevertheless, functional validation, particularly through lineage tracing and genetic perturbation *in vitro* and *in vivo*, remains essential for confirming candidate regulators and inferred developmental relationships. Despite these advances, several fundamental biological questions remain unresolved.

### Heterogeneity and developmental origin of AnSCs

Although single-cell analyses have identified multiple stem/progenitor populations within regenerating antlers, their developmental origins and lineage relationships throughout successive antler cycles remain incompletely resolved. In particular, whether cranial neural crest-derived progenitors directly contribute to antler generation and regeneration remains an open question. Future studies integrating spatial multi-omics with *in vivo* lineage tracing, including genetic fate mapping and CRISPR-based lineage recording (Fathi et al., 2026; Winter et al., 2025), will be essential for reconstructing developmental hierarchies and resolving the spatial dynamics of AnSC populations in their native tissue context.

### Gene regulatory networks governing antler development

Recent single-cell studies have identified multiple signaling pathways involved in antler generation, including AR-RXFP2, Wnt, angiogenic, and immune-associated pathways. However, how these pathways are integrated into dynamic gene regulatory networks (GRNs) that coordinate stem cell activation, lineage commitment, and tissue remodeling remains largely unknown. Future studies should combine single-cell transcriptomics, chromatin accessibility profiling, and perturbation-based approaches to reconstruct context-specific GRNs and identify key transcription factors controlling cell fate. Integrating computational network inference with functional validation will provide a systems-level understanding of how antler formation are initiated and maintained.

### Mechanisms of osteogenic transdifferentiation

The identification of a PHEX^+^ intermediate cell population suggests the existence of a hybrid ossification pathway linking hypertrophic chondrocytes to osteoblasts. However, whether these cells represent stable intermediates remains unresolved. Future studies should move beyond trajectory-based inference toward direct functional validation using lineage tracing together with *ex vivo* or 3D cartilage-bone organoid models (Bai et al., 2024) that recapitulate osteochondral transition. Time-resolved single-cell multi-omics combined with live imaging will further enable dynamic characterization of cell type transitions during hybrid ossification.

### Assembly of the regenerative vascular and immune niche

Rapid antler growth depends on the coordinated establishment of a specialized niche composed of endothelial, perivascular, stromal, immune, and stem/progenitor cells. Although recent studies have begun to reveal the cellular interactions underlying vascular development, the mechanisms coordinating vascular remodeling with immune regulation and stem/progenitor cell activation remain poorly understood. Future work integrating spatial transcriptomics, lineage tracing, and vascular organoid or microfluidic “antler-on-chip” systems will provide mechanistic insights into how these cellular compartments collectively support rapid growth.

### Metabolic reprogramming supporting rapid growth

The extraordinary growth rate of antlers requires extensive metabolic adaptation to sustain rapid proliferation, chondrogenesis, angiogenesis, and ossification, yet the underlying metabolic programs remain largely unexplored. Future studies should integrate spatial metabolomics, single-cell metabolic profiling, and stable isotope tracing (Yuan et al., 2019) with transcriptomic data to define metabolic fluxes within distinct growth niches. Such approaches will clarify how metabolic networks coordinate stem/progenitor activation, lineage transitions, and mineralization while maintaining exceptionally rapid yet non-tumorigenic tissue growth.

### Cross-species regenerative frameworks and translational applications

As the only mammalian organ capable of complete epimorphic regeneration, antlers provide a unique model for regenerative biology and translational medicine. Future efforts should prioritize the establishment of standardized multi-omics reference atlases and unified annotation pipelines to enable systematic comparisons with other regenerative systems, including digit tip, craniofacial tissues, dental pulp, and skeletal repair models. Integrating these comparative datasets with stem cell and organoid platforms will facilitate the translation of antler-derived regenerative principles into biomaterial design, tissue engineering, and regenerative medicine.
